# A Comprehensive Review of the Management of Light-Chain (AL) and Transthyretin (ATTR) Cardiac Amyloidosis

**DOI:** 10.31083/RCM42609

**Published:** 2025-12-18

**Authors:** Ahmad Alkhatib, Rama Alashqar, Ala W. Abdallah, Husam Abu-Nejim, Amer Hammad, Own Khraisat, Ahmed Sami Abuzaid

**Affiliations:** ^1^Department of Internal Medicine, MedStar Health Georgetown University, Baltimore, MD 21237, USA; ^2^Department of Internal Medicine, MedStar Health Georgetown University, Washington, DC 20007, USA; ^3^Department of Internal Medicine, Kirk Kerkorian School of Medicine at University of Nevada Las Vegas, Las Vegas, NV 89106, USA; ^4^Department of Internal Medicine, The Warren Alpert Medical School of Brown University, Providence, RI 02903, USA; ^5^Department of Internal Medicine, Englewood Health Medical Center, Englewood, NJ 07631, USA; ^6^Department of Cardiovascular Disease, Alaska Heart and Vascular Institute, Anchorage, AK 99508, USA

**Keywords:** amyloidosis, management, cardiac care, heart failure

## Abstract

Cardiac amyloidosis, once considered a rare and untreatable disorder, is now increasingly recognized as a significant cause of heart failure, particularly in older adults. The two most clinically relevant subtypes of cardiac amyloidosis—immunoglobulin light-chain amyloidosis (AL) and transthyretin-related amyloidosis (ATTR)—differ in pathogenesis, natural history, and management strategies, thereby necessitating a tailored approach to diagnosis and therapy. Advances in multimodality cardiac imaging, including echocardiography, cardiac magnetic resonance, and nuclear scintigraphy, have enabled earlier detection and improved differentiation between subtypes. Management of AL centers on rapid initiation of plasma cell-directed therapies to suppress light-chain production, with autologous stem cell transplantation and novel chemotherapeutic regimens improving survival. In contrast, ATTR management focuses on stabilizing or reducing transthyretin deposition through disease-modifying agents, such as stabilizers, gene-silencing therapies, and emerging fibril-disrupting approaches. Supportive care, including guideline-directed heart failure therapies and arrhythmia management, as well as advanced therapies such as transplantation, remains essential across both subtypes, albeit with unique considerations due to amyloid-related hemodynamics. This review synthesizes current evidence on the diagnosis and treatment of AL and ATTR, highlights recent therapeutic breakthroughs, and discusses ongoing challenges in optimizing patient outcomes, from equitable access to therapies to the integration of multidisciplinary care.

## 1. Introduction

Cardiac Amyloidosis is a type of infiltrative cardiomyopathy caused by extracellular myocardial deposition of amyloid fibril. This accumulation interferes with myocyte function, leading to restrictive filling physiology, with both diastolic and systolic heart failure. Additionally, amyloid deposits can disrupt electrical conduction causing arrhythmias, and infiltrate the coronary microvasculature, contributing to cardiac ischemia [1].

The two primary types of cardiac amyloidosis are light-chain amyloidosis (AL) and transthyretin (ATTR) amyloidosis. In AL, misfolded monoclonal immunoglobulin light chains from abnormal clonal proliferation of plasma cells form amyloid fibrils that deposit in the myocardium. In ATTR, misfolded transthyretin proteins aggregate to form amyloid fibrils that deposit in the myocardial interstitial space. ATTR can be inherited as an autosomal dominant trait caused by pathogenic variants in the transthyretin gene *TTR (ATTRv)* or by the deposition of wtATTR (wild-type transthyretin protein) which is mainly due to aging [2].

Recent data highlights that cardiac amyloidosis remains underdiagnosed. Efforts have concentrated on improving early detection and advancing novel therapies, as treatment is most beneficial in the disease’s early stages. However, there are still significant challenges in treating this condition due to its complexity and limited treatment options. In this review, we aim to review the current and future therapeutics reported in the literature for treatment of both AL and ATTR amyloidosis [3].

## 2. Guidelines Directed Medical Therapy in Cardiac Amyloidosis 

The physiology of restrictive left ventricular (LV) filling and reduced stroke volume in cardiac amyloidosis makes the use of conventional heart failure therapies challenging and requires a tailored approach [2]. There is a lack of studies concerning the efficacy of goal directed medical therapy as they are usually poorly tolerated given the disease pathophysiology. Expert consensus advises caution with the use of standard heart failure therapies in cardiac amyloidosis patients [4]. The vasodilatory effects of angiotensin receptor-neprilysin inhibitors (ARNi), angiotensin-converting enzyme inhibitors (ACEi), and angiotensin II receptor blockers (ARBs) may exacerbate hypotension, especially in the presence of amyloid-associated autonomic dysfunction and are poorly tolerated [5]. Furthermore, due to dependence on the chronotropic response, beta blockers were suggested to be poorly tolerated in patients with cardiac amyloidosis, especially in cases with overt restrictive filling [2, 6].

Despite expert consensus guidelines advising caution with the use of standard heart failure therapies, studies by Aimo *et al*. [7] and Yan *et al*. [8], showed that a significant proportion of patients with cardiac amyloidosis were prescribed standard heart failure therapy and tolerated them well, in the absence of contraindications. A retrospective analysis by ​​Ioannou *et al*. [9] has shown mortality benefit with low dose beta-blocker in patients with a reduced left ventricular ejection fraction of <40%. Mineralocorticoid receptor antagonists (MRAs) were also shown to be independently associated with improved mortality, regardless of the ejection fraction.

Despite sodium glucose cotransporter 2 inhibitors (SGLT-2I) being associated with improved mortality in patients with heart failure with reduced and preserved ejection fraction, cardiac amyloidosis patients were not part of the phase III trials [10, 11, 12, 13]. Despite this, studies have shown good tolerance to SGLT-2I patients with cardiac amyloidosis [14, 15]. Moreover, an observational study by Porcari *et al*. [15] showed lower all-cause and cardiovascular mortality, as well as cardiovascular hospitalization in patients with ATTR cardiac amyloidosis being treated with SGLT-2I. As for AL cardiac amyloidosis, a study by Lang *et al*. [16] showed reduced diuretic doses, and N-terminal pro–B-type natriuretic peptide (NT-proBNP) levels in patients on SGLT-2I. Further prospective studies are needed to confirm the efficacy and safety of conventional heart failure therapies in cardiac amyloidosis. The cornerstone of treatment in this population relies on disease modifying therapy and symptomatic management.

## 3. The Management of AL Cardiac Amyloidosis 

The management of cardiac amyloid AL requires a multidisciplinary approach, involving hematology-oncology specialists as well as cardiologists given the fact that the degree of cardiac involvement is the most important prognostic factor in AL amyloidosis, and forms the basis of widely used staging systems [17]. Circulating cardiac biomarkers, specifically NT-proBNP and cardiac troponin, correlate with myocardial amyloid burden and are the basis of those staging systems [17]. The Mayo Clinic 2004 staging model stratified risk into three stages based on a troponin T cutoff of 0.035 µg/L and an NT-proBNP cutoff of 332 ng/L. An updated Mayo 2012 system added the difference between involved and uninvolved light chains (dFLC ≥180 mg/L) as a third risk factor, defining Stage I with no risk factors through Stage IV with all three risk factors present [17]. In this 4-tier system, Stage IV carries a median survival under 6 months if untreated [18]. The European modification in 2016 further subdivided the highest-risk group by an NT-proBNP cut off 8500 ng/L with Stage IIIb being higher and Stage IIIa being lower. This was mainly due to​ the extreme mortality of Stage IIIb disease (median of ~4 months)​ [19, 20]. In practice, these staging systems guide therapy intensity. Patients with advanced cardiac biomarkers (Stages III/IV) are frail and may require adjusted dosing or alternate strategies. Other factors portending a worse prognosis include very low systolic blood pressure (autonomic neuropathy), multiple organ involvement (renal, hepatic, etc.), and poor performance status​. Staging at diagnosis is essential for risk stratification and helps predict tolerance to aggressive treatments like stem cell transplant [21].

### 3.1 First Line Therapies 

Frontline treatment of AL amyloidosis centers on rapid eradication of the pathogenic plasma cell clone. Based on recent phase III evidence, the combination of daratumumab (anti-CD38 monoclonal antibody) with cyclophosphamide, bortezomib, and dexamethasone (Dara-CyBorD) is now established as the standard initial regimen for most patients​. In the ANDROMEDA trial, adding daratumumab to CyBorD tripled the rate of complete hematologic response and led to significantly higher 6-month cardiac and renal response rates compared to CyBorD alone [22]. Based on these results, Dara-CyBorD achieved regulatory approval as first-line therapy and is recommended in consensus guidelines for newly diagnosed AL amyloidosis [22].

An alternative regimen is a bortezomib-based regimen that remains highly effective. A Real-world series of more than 900 patients treated with bortezomib combinations (typically CyBorD) reported overall hematologic response rates of ~65%, complete response of ~25%, and median overall survival around 6 years [23]. Bortezomib’s proteasome inhibition produces rapid light-chain reduction and is generally well-tolerated, though neuropathy can be dose-limiting. For patients with significant pre-existing peripheral neuropathy, guidelines suggest avoiding neurotoxic agents. One option is a non-bortezomib regimen such as melphalan plus dexamethasone combined with daratumumab [24]. Melphalan/dexamethasone (MDex) was the prior first-line therapy before bortezomib’s era and can still induce hematologic responses, though typically slower and lower in depth than bortezomib-based therapy [17]. Notably, melphalan is now mostly reserved for transplant conditioning or for patients who cannot take bortezomib. In any case, virtually all patients with systemic AL amyloidosis require prompt therapy at diagnosis. Observation is only appropriate for the rare truly localized AL or incidental clonal findings without organ involvement [25].

#### 3.1.1 Autologous Stem Cell Transplant (ASCT)

High-dose melphalan followed by ASCT can produce long-term remissions and is an important first-line consideration in eligible patients. Transplant offers the deepest clonal responses, which can translate into prolonged organ function improvement and survival. However, only a minority (~20%) of AL patients qualify for ASCT at diagnosis [25]. Selection criteria are strict due to the treatment toxicity. Patients should typically be <65–70 years old, with preserved performance status and limited critical organ involvement. Advanced cardiac amyloidosis in particular increases transplant risk. Overt heart failure, significant arrhythmias, or very high NT-proBNP are markers for poor candidacy [26]. Consensus transplant guidelines list uncontrolled arrhythmias, decompensated heart failure, untreated pleural effusions, refractory hypotension, or multi-organ failure as contraindications to ASCT​ [27]. Still, ASCT should be undertaken in centers with amyloidosis expertise, as peri-transplant morbidity is higher than in myeloma. An individualized approach is key, as for some stage I–II patients, upfront ASCT is favored, whereas others receive full courses of Dara-CyBorD and reserve transplant for consolidation if a deep response is not reached. Emerging data also suggest that sequential organ transplant and ASCT can be lifesaving in extreme cases. Such an approach has been successfully done in select patients, though it requires careful timing and multidisciplinary coordination [4].

#### 3.1.2 Monitoring Treatment Response

AL amyloidosis requires frequent monitoring to assess hematologic response. Hematologic response is tracked with the same tools used in plasma cell dyscrasias with serum free light chain assays, quantitative immunoglobulins, and serum/urine immunofixation. The goal is complete eradication of the pathogenic light chain clone, as deeper hematologic responses strongly correlate with improved organ outcomes and survival [28, 29]. Complete response (CR) indicates negative serum and urine immunofixation, and normalization of the free light chain ratio. This implies elimination of the plasma cell clone to an undetectable level. Very good partial response (VGPR) implies a difference between involved and dFLC of <40 mg/L​. In practice, this equates to a >90% reduction in circulating light chain burden from baseline in most patients. Partial response (PR) is >50% reduction in dFLC from baseline. No Response is anything less than PR. CR is the ideal target, as it is associated with the highest likelihood of organ improvement. Light chain levels are typically checked monthly during therapy. In AL, unlike multiple myeloma, bone marrow biopsies to confirm CR are not recommended unless considering a clinical trial or transplant. Blood and urine markers usually suffice to declare a response. Light chain levels are typically checked monthly during therapy.

### 3.2 Relapse and Second Line Therapies

Despite optimal first-line treatment, a subset of AL amyloidosis patients will have persistent disease or experience relapse. Management of relapsed/refractory AL is often extrapolated from multiple myeloma, although data specific to AL are emerging. If a patient did not receive one of the major drug classes initially, introducing that class is a common strategy. There is no consensus on a single second line agent.

Bortezomib, a proteasome inhibitor (PI), can be reused if the initial response was good. Otherwise, next-generation PIs like carfilzomib and ixazomib have been explored. Ixazomib (oral PI) plus dexamethasone showed activity in early-phase studies and offers an all-oral regimen for frail patients​. Ongoing trials are assessing ixazomib in combinations (e.g., with lenalidomide or venetoclax).

Lenalidomide and pomalidomide are immunomodulators and can be combined with dexamethasone (with or without cyclophosphamide). They have moderate efficacy in AL. These agents can suppress the plasma cell clone but often cause fluid retention or cardiac side effects, so doses are started low. They are typically considered after PI-based therapy.

Daratumumab is a monoclonal antibody. If it was not used first-line, it is highly effective in relapse and can be given either alone or with other agents (trials show daratumumab monotherapy yields ≈40% responses in AL). Another anti-CD38 antibody, isatuximab, is in clinical trials for refractory AL amyloidosis​. Additionally, elotuzumab (targeting SLAMF7, a member of signaling lymphocyte activation molecule family of proteins) is being tested in combination with lenalidomide/dex (a Phase II trial for relapsed AL)​. Patients who didn’t undergo upfront ASCT might be evaluated for transplant in first relapse if their performance status has improved and disease is chemoresponsive.

A significant proportion (50–60%) of AL amyloidosis plasma cell clones have the translocation t(11;14), which confers high B-cell lymphoma-2 (BCL-2) expression. Venetoclax, a BCL-2 inhibitor, has shown remarkable efficacy in this subset. Although not yet Food and Drug Administration (FDA)-approved for AL, venetoclax is used off-label in relapsed cases with t(11;14) and has demonstrated high hematologic response rates and good tolerability [30]. Venetoclax is also being investigated in clinical trials, including in upfront combination with daratumumab for newly diagnosed AL​. Another novel agent under study is selinexor (an exportin-1 or XPO1 inhibitor), which has shown some activity in advanced AL amyloidosis and is in an early-phase trial for relapsed disease​.

### 3.3 Investigational Therapies

Encouraging progress is being made in therapies that go beyond suppressing light-chain production, aiming to directly target amyloid deposits or new biology. A major area of research is fibril-directed monoclonal antibodies, which seek to bind amyloid deposits in tissues and promote their clearance by the immune system. Two leading candidates in late-stage development are CAEL-101 and birtamimab.

CAEL-101: A chimeric IgG1 monoclonal antibody that targets kappa and lambda light-chain fibrils, CAEL-101 is designed to opsonize amyloid deposits and stimulate phagocytic clearance [31]. In phase I/II studies, CAEL-101 was well tolerated and showed organ biomarker improvements when added to standard chemotherapy, especially in cardiac AL patients. Two phase III trials of the CARES study are underway, evaluating CAEL-101 plus standard of care vs placebo in newly diagnosed AL patients with advanced cardiac involvement (Mayo stage IIIa and IIIb) [20]. Unfortunately, phase III trials did not meet the primary endpoint, a combination of the time to all-cause mortality and the frequency of cardiovascular hospitalizations, in the overall enrolled population. However, in a prespecified group, Anselamimab demonstrated highly clinically meaningful improvements in both survival and cardiovascular hospitalization outcomes compared to placebo. The future of the development of Anselamimab remains uncertain.

Birtamimab is another monoclonal antibody targeting deposited light-chain amyloid. An initial phase III trial (VITAL) was terminated early for futility in an unselected AL population. However, post-hoc analysis revealed a striking survival benefit in the sickest subset – Mayo stage IV patients. Among these advanced cardiac patients, those who received birtamimab had 74% 9-month survival versus 49% on placebo​. They also showed a better quality of life and 6-minute walk​. This signal suggests that removing amyloid deposits may be most impactful in patients at highest risk of early death. Consequently, a new phase III trial (AFFIRM-AL) has been launched, enrolling only Mayo stage IV patients to confirm whether adding birtamimab to standard therapy improves survival in this group. However, the AFFIRM-AL did not meet the primary end point of all-cause mortality, and the secondary endpoints did not achieve statistical significance. This has led to the discontinuation of the program.

## 4. The Management of ATTR Cardiac Amyloidosis and Disease Modifying Therapy

### 4.1 Transthyretin Stabilizers

Transthyretin stabilizers are small molecules designed to stabilize the tetrameric structure of the transthyretin (TTR) protein, thereby preventing its dissociation into monomers, which is the rate-limiting step in the formation of amyloid fibrils [32]. Targeted therapies have focused on small molecules that stabilize the tetramer, inhibiting tetramer dissociation and amyloidogenesis.

Tafamidis was first discovered to bind transthyretin at the thyroxine binding sites, stabilizing the tetrameric structure of TTR and reducing dissociation and deposition of amyloid fibrils in the myocardium [32]. Efficacy and safety of Tafamidis were investigated in the ATTR-ACT trial, which investigated both types of transthyretin amyloid cardiomyopathy and showed that Tafamidis was associated with a reduction in all-cause mortality, hospitalizations due to cardiovascular events and in the decline of functional capacity and quality of life [33]. The US Food and Drug Administration approved Tafamidis for use in ATTR cardiomyopathy (ATTR-CM) in 2019.

Acoramidis, also known as AG10, is another high-affinity TTR stabilizer and binds more strongly to the thyroxine binding sites than does tafamidis. It mimics the coinheritance of the TTR *T119M* mutation that provides natural stabilization of TTR [34]. The efficacy and safety of Acoramidis were studied in the ATTRibute-CM trial, which showed an overall reduction in all-cause mortality and hospitalizations associated with cardiovascular disease [35]. Further open-label extension study showed sustained clinical benefit and confirmed safety profile over 42 months of follow-up [36]. The US FDA approved Acoramidis for use of ATTR-CM in 2024.

Dinflunisal is a non-steroidal anti-inflammatory drug (NSAID) that stabilizes the TTR tetramer. Retrospective analysis showed measurable differences in cardiac structure and function parameters, such as TTR concentration and left atrial volume index, after one year of treatment [37]. Further reviews showed that it is associated with a reduction in mortality and a decrease in the number of orthotopic heart transplants in patients with ATTR-CM [38]. Another study found that diflunisal treatment in patients with early-stage wild-type ATTR amyloid cardiomyopathy (wtATTR-CM) was associated with increased survival and a reduction in overall mortality [39]. Although Diflunisal has shown potential benefits, it is not yet approved for use and it is used off-label. Long-term and randomized prospective studies are needed to confirm these findings.

### 4.2 Suppression of TTR Production

Therapies suppressing TTR synthesis include liver transplantation, gene silencing therapies (small interfering RNA [siRNA]) and antisense oligonucleotides (ASO), and gene-editing technology, CRISPR/Cas9. TTR stabilizers are currently the leading therapies for treating ATTR-CM, including selective agents that are in the spotlight.

Since TTR is primarily produced in the liver, transplantation can reduce the production of mutant TTR variants. Prior to 2018, liver transplantation was the primary treatment for hereditary ATTR, aiming to reduce the production of mutant transthyretin. Similarly, heart transplantation, with concurrent liver transplantation, was considered for patients with ATTR-CM. However, these surgical interventions were often limited by factors such as advanced age and the complexities associated with multiorgan transplantation. While still available, the development of effective drug therapies in recent years has provided alternative treatment options that offer improved outcomes and quality of life for patients with ATTR amyloidosis [40].

siRNAs are synthetic, double-stranded RNA molecules that mediate gene silencing through the RNA interference pathway. They act at the post-transcriptional level, targeting specific messenger RNA (mRNA) for degradation, thereby preventing translation into protein. Unlike other gene-silencing methods like antisense oligonucleotides, siRNAs require 100% complementarity to the target sequence, making them highly specific [41]. In the context of TTR amyloidosis, siRNA therapeutics target the *TTR* gene, which codes for the TTR protein. Two siRNA drugs have been developed for this purpose, Vutrisiran and Patisiran.

Vutrisiran is a subcutaneously administered RNA interference therapeutic agent that inhibits the hepatic synthesis of both the wild and variant types of TTR messenger RNA [42]. Its molecular design incorporates phosphorothioate linkages at the 5^′^ end of its siRNA, along with a triantennary N-acetylgalactosamine (GalNAc) ligand, which targets the asialoglycoprotein receptor on hepatocytes. Also, an increased proportion of 2^′^-O-methyl nucleotides; all these molecular chemical enhancements contribute to a greater structural stability and potency compared to earlier RNA interference therapies. These structural optimizations enable the drug to be administered as infrequently as once every three months [43]. Vutrisiran was FDA-approved in June 2022 in the USA for the treatment of polyneuropathy of hereditary ATTR amyloidosis in adults after positive results from the HELIOS-A trial [44]. The HELIOS-B trial randomized 655 patients to receive either 25 mg of Vutrisiran or placebo via subcutaneous injection every 3 months for up to 36 months. Patients who were already taking tafamidis at the start of the trial were allowed to continue it, and randomization was stratified to an overall population (Tafamidis at baseline + Vutrisiran) and a monotherapy population (Vutrisiran only). Vutrisiran demonstrated a significant clinical benefit over placebo in both the overall and monotherapy groups. The treatment group experienced improvements in the primary endpoint, a composite of all-cause mortality and recurrent cardiovascular events, as well as in all major secondary endpoints. NT-proBNP levels, a marker of disease progression-remained stable in the Vutrisiran group but increased in the placebo arm. Vutrisiran also resulted in a mean reduction in transthyretin levels of approximately 80%. Vutrisiran was recently approved by the FDA in March 2025. However, the study was limited in its representation of certain subgroups, with only 7.5% women, 15.6% non-White participants, and 11.6% carrying variant TTR mutations; indicating a need for better subgroup representation in future studies [42].

Patisiran, a siRNA that inhibits hepatic synthesis of TTR. Patrisiran was the first siRNA developed for ATTR amyloidosis and was FDA approved for the treatment of hereditary TTR peripheral neuropathy. It is a liposome formulation consisting of a slightly modified siRNA encapsulated with lipid excipients to ensure delivery into the hepatocyte. The siRNA was slightly modified to improve its stability and to avoid off-target effects [45]. Once it reaches the hepatocyte cytoplasm, the double-stranded siRNA splits into single stranded RNAs that bind to complementary mRNA, which results in activation of the Argonaute slicer protein, that degrades the mRNA thus inhibiting TTR synthesis [9, 45]. Patisiran is administered intravenously every three weeks, with a recommended dose of 0.3 mg/kg, reaching steady-state concentrations within 24 weeks. To mitigate infusion-related reactions, patients typically receive pre-medication with antihistamine, corticosteroid and oral acetaminophen beforehand [46]. Clinical trials have shown it improves neuropathy and quality of life in hereditary ATTR amyloidosis and based on the APOLLO-A trial, Patisiran gained FDA approval for treatment of hereditary ATTR with polyneuropathy and supported its potential role in ATTR-CM [47]. The APOLLO-B trial evaluated Patisiran’s effects on functional status and quality of life in patients with ATTR-CM over 12 months and confirmed the significant benefit of Patisiran in preserving functional capacity and quality of life in patients with ATTR-CM. This is the first study to show the efficacy of RNA silencing therapy in ATTR-CM. Despite the positive findings of the study, the drug was not approved by the FDA. An open-label extension to the APOLLO-B trial is currently underway to assess Patisiran’s long-term safety, survival, and hospitalizations [48].

Inotersen is an ASO that reduces hepatic production of TTR. NEURO-TTR was a clinical trial that enrolled patients with hereditary ATTR with polyneuropathy in the presence or absence of ATTR-CM. The study demonstrated significant improvement in neuropathy symptoms and better quality of life when compared to placebo [49], which resulted in gaining FDA approval for treatment of hereditary ATTR with polyneuropathy. A prespecified open-label extension of the NEURO-TTR trial demonstrated that Inotersen maintained quality of life over 2–3 years, with no new safety issues, supporting its benefit in hereditary transthyretin amyloidosis with polyneuropathy [50]. This trial however was not powered to evaluate cardiac outcomes to assess for treatment of ATTR-CM, it was noted that global longitudinal strain on echocardiography did not differ significantly between groups [49].

Eplontresen is another ASO with similar design to Inotersen and is conjugated to GalNAc which results in selective uptake by hepatocytes [51], which limits off-target interactions in non-hepatic tissues such as platelets and glomeruli seen in Inotersen. NEURO-TTRansform was a clinical trial that enrolled patients with hereditary ATTR amyloidosis polyneuropathy and demonstrated a significant decrease in serum TTR concentration, neuropathy symptoms and better quality of life compared to placebo [52]. Further subgroup analysis of the NEURO-TTRansform trial patients with ATTRv-PN who switched from Inotersen to Eplontersen, to investigate the safety and efficacy of switching from Inotersen to Eplontersen. Results showed further reduced serum TTR, halted disease progression, stabilized quality of life, restored platelet count, and improved tolerability, without deterioration in nutritional status. This supports that Eplontersen offers a more favorable safety and tolerability profile [53]. Posthoc analysis of NEURO-TTRansform trial’s ATTR-CM subpopulation showed stable or improved cardiac structure and function, including significant improvement in LVEF and stroke volume. These results prompted further assessment of cardiac efficacy, CARDIO-TTRansform is an ongoing phase III clinical trial assessing the safety and cardiac efficacy, mortality and recurrent cardiovascular events of Eplontersen compared to placebo. Results of this trial are expected to be reported in 2025.

CRISPR-Cas9, which means clustered regularly interspaced short palindromic repeats and associated Cas9 endonuclease system, is made up of a lipid nanoparticle (LNP) that carries a single guide RNA which targets the *TTR* gene and Cas9 mRNA that gets translated to the Cas9 endonuclease. This system allows the TTR genome to be altered, resulting in a targeted double-stranded DNA cleavage. NTLA-2001 is a novel *in vivo* gene-editing therapy that gets taken up by low density lipoprotein (LDL) receptors on hepatocytes followed by endocytosis. Thereafter, CRISPR-Cas9 system is utilized to knock out the *TTR* gene in liver cells, preventing downstream TTR protein production which would reduce TTR amyloid fibril deposition. The first ever human clinical trial of NTLA-2001 preliminary results showed a significant reduction in serum TTR protein levels in phase I trial after receiving a single dose at 28 days. It was also noted to be dose-dependent reduction in TTR levels; showing a mean reduction of circulating TTR by 51.66% in the group that received a dose of 0.1 mg/kg and a mean reduction of circulating TTR by 86.66% in the group that received a dose of 0.3 mg/kg at 28 days [54]. Further analysis of the trial upon completion of enrollment of Phase I showed a mean reduction of 89% at 28 days that was sustained at 90% for up to 2 years afterwards [55]. However, long-term efficacy and adverse effects are yet to be assessed, including sustainability of TTR reduction in the long term. MAGNITUDE trial is a phase III clinical trial to evaluate the efficacy and safety of a single dose of NTLA-2001 compared to placebo in participants with ATTR-CM which was launched on March 18, 2024. CRISPR-Cas9 offers a potential one-time treatment by permanently disrupting the *TTR* gene, thus halting amyloid formation at its source.

### 4.3 Monoclonal Antibodies

NI006 (also known as ALXN2220) is a recombinant human monoclonal antibody, similar characteristics to IgG1, designed to selectively bind misfolded TTR and promote its clearance via antibody-mediated phagocytosis without binding to native TTR. In the Phase 1 NI006-101 trial, 40 patients with wild-type or variant ATTR cardiomyopathy and chronic heart failure were randomized (2:1) to receive ascending doses of NI006 (0.3–60 mg/kg) or placebo every 4 weeks for 4 months, followed by an 8-month open-label extension (OLE). The treatment was well tolerated, with no drug-related serious adverse events, and no anti-drug antibodies were detected throughout the trial. The median extracellular volume (ECV) on cardiac magnetic resonance (CMR) decreased from 59.4% to 41.6%, and median heart-to-whole-body uptake ratios on scintigraphy declined from 5.7% to 2.5% at 12 months. In addition, biomarkers such as NT-proBNP and troponin levels demonstrated a significant decrease [56]. A prolonged OLE study evaluated long-term safety and efficacy beyond 12 months, with 23 participants continuing up to a median of 20 infusions over 125 weeks. All patients were up titrated to 30 mg/kg. Based on results reported in an abstract, the intervention was well tolerated and cardiac imaging and cardiac biomarker data indicated continued treatment effects beyond 12 months. However, the information is derived from an abstract, which may have limitations compared to a full-text article. These results provide proof-of-concept for monoclonal antibody-mediated amyloid depletion in ATTR-CM and support the advancement of ALXN2220 to Phase 3 evaluation, DepleTTR-CM trial, that is currently ongoing.

PRX004 is a humanized monoclonal antibody that selectively binds misfolded transthyretin without interacting with the native tetrameric form, allowing it to inhibit amyloid fibril formation of TTR while preserving normal TTR function. It also promotes immune-mediated clearance of amyloid deposits through phagocytosis. In a Phase 1 open-label dose-escalation study involving 21 patients with hereditary ATTR, PRX004 was administered intravenously at doses ranging from 0.1 to 30 mg/kg every 28 days. The treatment was well tolerated, with no dose-limiting toxicities observed and no maximum tolerated dose reached. PRX004 demonstrated pharmacodynamic activity with a dose-dependent reduction in free misTTR levels by up to 50.2%. The study concluded that PRX004 was well tolerated in patients with ATTRv amyloidosis and demonstrated potential clinical activity. Although the long-term extension phase was initiated, it was terminated early due to the COVID-19 pandemic [57]. The antibody is now under further development as NNC6019 and is being evaluated in a Phase 2 randomized, double-blind, placebo-controlled trial is currently ongoing to evaluate two doses (30 mg/kg and 100 mg/kg) in 99 patients with wild-type or hereditary ATTR-CM [58]. The primary endpoints include change in 6-minute walk distance and NT-proBNP levels, while secondary outcomes assess cardiac structure and function, hospitalizations, Kansas City Cardiomyopathy Questionnaire (KCCQ) scores, and all-cause mortality. This study will help define the optimal dosing for a planned Phase 3 trial and further clarify NNC6019’s role in treating ATTR-CM.

Doxycycline and TUDCA (tauroursodeoxycholic acid) are an experimental combination regimen that can cause transthyretin disruption. Doxycycline disrupts amyloid fibrils, while TUDCA inhibits apoptosis and aids in protein folding. Preliminary studies suggest this combination may reduce amyloid deposits and improve cardiac function.

While Tafamidis, Acoramidis and Vutrisiran are approved for treating ATTR-CM, other therapies like diflunisal, gene silencers, gene editing and monoclonal antibodies are still under investigation or approved for specific ATTR subtypes. The combination of doxycycline and TUDCA is experimental, and its efficacy requires further validation. Treatment strategies should be individualized based on patient-specific factors and disease characteristics. The emergence of these new disease modifying therapies for ATTR-CM raises new challenges in comparing efficacy, appropriate use and determining whether combining these agents or sequentially delivering them. The baseline characteristics of the patients differed among ATTR-ACT trial of Tafamidis, HELIOS-B trial of Vutrisiran, and the ATTRibute-CM trial of Acoramidis, making it impossible to compare the overall health benefits of the three agents. Further studies are needed to clarify the most effective sequence or combination of these FDA-approved agents. A summary of the drugs and their mechanism of actions, trial name, and FDA approval date can be found in Table 1 (Ref. [33, 35, 37, 38, 39, 42, 44, 47, 48, 49, 52, 54, 56, 57, 58]).

**Table 1.** **Disease modifying therapy of ATTR amyloidosis**.

Drug name	Mechanism of action	Clinical trial	Trial outcome	FDA approval
Tafamidis	Transthyretin tetramer stabilizer; binds thyroxine binding sites	ATTR-ACT (Maurer *et al*. [33], 2018)	Lower all-cause mortality and cardiovascular hospitalizations compared to placebo	Approved 2019 for ATTR-CM
Acoramidis (AG10)	TTR stabilizer mimicking T119M mutation; stronger binding than tafamidis	ATTRibute-CM (Gillmore *et al*. [35], 2024)	A four-component hierarchical primary endpoint combining all-cause mortality, cardiovascular hospitalization, NT-proBNP change, and 6-minute walk distance showed a statistically significant benefit of Acoramidis over placebo	Approved 2024 for ATTR-CM
Diflunisal	NSAID that stabilizes TTR tetramer	Multiple retrospective studies (Lohrmann *et al*. [37], Ibrahim *et al*. [38], Siddiqi *et al*. [39])	wtATTR–CM showed improved survival with Diflunisal compared to placebo	Not FDA approved; off-label use
Patisiran	siRNA targeting hepatic TTR mRNA; delivered via lipid nanoparticles	APOLLO-A (Adams *et al*. [47], 2018)	Improved functional capacity and quality of life	Approved 2018 for hATTR-PN; Not approved for ATTR-CM
		APOLLO-B (Maurer *et al*. [48], 2023)		
Vutrisiran	Subcutaneous GalNAc-siRNA targeting TTR mRNA	HELIOS-A (Adams *et al*. [44], 2023)	HELIOS-B trial showed improved mortality and lower rates of cardiovascular events	Approved 2022 for hATTR-PN;
		HELIOS-B (Fontana *et al*. [42], 2025)		Approved 2025 for ATTR-CM
Inotersen	Antisense oligonucleotide inhibiting hepatic TTR production	NEURO-TTR (Benson *et al*. [49], 2018)	Slower progression of neuropathy	Approved 2018 for hATTR-PN; Not for ATTR-CM
			Stabilization of cardiovascular parameters	
Eplontersen	GalNAc-conjugated ASO with enhanced hepatocyte targeting	NEURO-TTRansform (Coelho *et al*. [52], 2023)	Improved neuropathy and quality of life	Not yet approved; ongoing trial for ATTR-CM
		Undergoing CARDIO-TTRansform trial		
NTLA-2001	*In vivo* CRISPR-Cas9 gene editing to permanently disrupt *TTR* gene	Phase I (Gillmore *et al*. [54], 2021); Ongoing MAGNITUDE trial	Mean reduction of serum TTR levels of 90% by 28 days in phase I	Not yet approved; Ongoing Phase III trial for ATTR-CM
NI006/ALXN2220	Recombinant human anti-ATTR antibody; targets TTR deposits	Phase I complete (Garcia-Pavia *et al*. [56], 2023);	Reduced cardiac tracer uptake on scintigraphy and decreased extracellular volume on cardiac magnetic resonance over a 12-month period in phase IB.	Not approved; in Phase III
		Phase III ongoing (DepleTTR-CM)		
			Reductions in NT-ProBNP and troponin levels in phase 1B.	
			Improvements in Kansas City Cardiomyopathy Questionnaire scores in phase IB.	
NNC6019/PRX004	Humanized monoclonal antibody targeting misfolded monomeric and aggregated TTR	Phase I (terminated due to COVID) (Suhr *et al*. [57], 2025)	Improvements in the Neuropathy Impairment Score (NIS) and global longitudinal strain in phase IB.	Not approved; in Phase II
		Phase II ongoing (Fontana *et al*. [58], 2022)		
Doxycycline + TUDCA	Disrupts fibrils (doxycycline); anti-apoptotic & protein folding aid (TUDCA)	Preliminary studies; no Phase 3 trials yet	Slower progression of neuropathy	Not approved; experimental
			Stabilized echocardiographic measures and cardiac biomarkers	

Approvals for ATTR-CM specifically are noted where applicable. Eplontersen: ongoing CARDIO-TTRansform trial aims to determine FDA eligibility for ATTR-CM. NTLA-2001: undergoing MAGNITUDE Phase III trial as a potential one-time CRISPR-based therapy. NI006/ALXN2220: ongoing DepleTTR-CM trial aims to determine FDA eligibility for ATTR-CM. hATTR-PN, hereditary ATTR with polyneuropathy; ATTR-CM, transthyretin amyloid cardiomyopathy; ATTR, transthyretin-related amyloidosis; TTR, transthyretin; NT-proBNP, N-terminal pro–B-type natriuretic peptide; siRNA, small interfering RNA; mRNA, messenger RNA; ASO, antisense oligonucleotides; NSAID, non-steroidal anti-inflammatory drug; FDA, Food and drug Administration.

### 4.4 Monitoring Treatment Response

Recent consensus from the European Society of Cardiology (ESC), Japanese Circulation Society (JCS), and American Society for Nuclear Cardiology (ASNC) recommends standardized, quantitative, and harmonized imaging protocols for both diagnosis and longitudinal monitoring. Positive scintigraphy (Perugini grade ≥2 or heart to contralateral ratio of ≥1.5) in the absence of a monoclonal protein is sufficient to establish the diagnosis noninvasively. In cases with equivocal uptake or concomitant monoclonal protein, further evaluation with cardiac magnetic resonance, endomyocardial biopsy, or genetic testing is recommended [59, 60]. Single photon emission computed tomography -computed tomography (SPECT-CT) has also been recently incorporated into the guidelines, particularly in the cases of ambiguous planar scans, guiding biopsy decisions, and confirming therapeutic response. Imaging findings should be integrated with cardiac biomarker results and echocardiography findings to improve diagnostic accuracy [59, 60].

In patients receiving TTR-stabilizers or silencing therapies, serial technetium-99m pyrophosphate (99mTc-PYP) scans have been increasingly recommended to monitor treatment response. Individualized imaging intervals, typically every 1–2 years, allow for the assessment of amyloid burden over time using quantitative imaging metrics such as heart-to-contralateral ratio (H/CL) and SPECT-derived standardized uptake values (SUV) [61].

## 5. The Management of Arrhythmias in Cardiac Amyloidosis

Atrial arrhythmias, ventricular arrhythmias, and conduction disease have all been well described in cardiac amyloidosis in both the ATTR and the AL subtypes [62, 63, 64, 65, 66, 67]. Generally, arrhythmias in patients with cardiac amyloidosis are poorly tolerated. Despite the prevalence of arrhythmias in patients with cardiac amyloidosis, guideline recommendations and high-quality evidence for management are scarce. The use and timing of implantable cardioverter defibrillators (ICD) devices in the prevention of sudden cardiac death remains disputable [68, 69]. Moreover, rate control therapies for atrial arrhythmias are poorly tolerated in this patient population [67, 70].

### 5.1 Atrial Arrhythmias

Multiple mechanisms are thought to contribute to the development of atrial arrhythmias in patients with cardiac amyloidosis. Extensive amyloid fibril deposition in the atrial walls leads to the alteration of normal tissue architecture with an increase in stiffness, altered contractility, and a restrictive filling pattern, promoting the occurrence of atrial arrhythmias, particularly atrial fibrillation (AF) [71, 72, 73, 74]. There is a positive correlation between amyloid fibril deposition and the presence of long-standing atrial fibrillation [75, 76].

The prevalence of atrial arrhythmias, especially atrial fibrillation, in cardiac amyloidosis is higher than the general population [62, 77]. Sanchis *et al*. [62] reported the prevalence of atrial fibrillation to be 44% in cardiac amyloidosis patients, as compared to 1% in the general population. The prevalence of atrial fibrillation has also been shown to be different among the different types of cardiac amyloidosis, with wild type ATTR (wtATTR) being most commonly associated with atrial fibrillation. Mints *et al*. [77] showed that the incidence of AF in a population of 146 individuals with wtATTR can be as high as 70%, while the study by Sanchis *et al*. [62] reported a prevalence of AF in wtATTR of 71%. Similar findings denoting the higher prevalence of AF in TTR cardiac amyloidosis were reported by Cyrille *et al*. [78] and Longhi *et al*. [79]. The higher prevalence of AF in the wtATTR groups has been attributed to the patient demographic being mainly comprised of elderly males with long standing heart failure [74].

Due to the restrictive physiology in patients with cardiac amyloidosis and the heart rate being the main driver of cardiac output, rate control is often challenging. Beta blockers, calcium channel blockers, and digoxin all have negative inotropic effects and depress the heart rate leading to poor tolerance in this patient population. They have been reported to cause hypotension and heart failure exacerbations [74, 80, 81, 82]. Digoxin has been associated with cardiac toxicity that cannot be excluded based on blood levels alone due to the drug binding to the amyloid fibrils prolonging its half-life [82, 83]. Starting at low doses, hemodynamic monitoring, frequent digoxin drug levels along with kidney function and electrolytes is required if Digoxin is to be trialed [83]. As with all cases of atrial fibrillation refractory to medical therapy, atrioventricular nodal ablation and permanent pacemaker implantation can be considered [84].

Many experts favor rhythm control strategies in AF in cardiac amyloidosis due to the poor tolerance of pharmacologic rate control. Amiodarone seems to be well tolerated in this patient population and could be the antiarrhythmic of choice [74]. A study performed by Donnellan *et al*. [85] has demonstrated that maintenance of normal sinus rhythm improved survival in patients with ATTR cardiac amyloidosis and atrial fibrillation. However, there was no difference in mortality when comparing antiarrhythmic therapy to rate-control agents, but rhythm control strategy was more effective in the maintenance of normal sinus rhythm in the first stages of the disease [85]. Similar findings on the lack of mortality benefit were previously reported by Mints *et al*. [77].

Anticoagulation remains one of the cornerstones in the management of atrial fibrillation [84]. The risk of stroke, cardiac thrombus and systemic embolization in cardiac amyloidosis is higher than that of the general population [74, 86]. This has been attributed to fibril deposition in the atrial wall, which leads to reduced atrial contractility with higher blood stasis, and endothelial dysfunction resulting in higher hypercoagulability [73, 87]. A case series done by Dubrey *et al*. [87] demonstrated thrombus formation in this patient population while in sinus rhythm. A study by Donnellan *et al*. [88] on a population of patients with ATTR cardiac amyloidosis demonstrated an incidence of 30% of left atrial appendage (LAA) thrombus, 26% of those who had LAA thrombus were already on systemic anticoagulation prior to diagnosis. Moreover, the study showed no proportional increase in thrombus formation in relation to the CHADS-VASc score, rendering it inapplicable in this patient population. This has led to the rise of an expert consensus to start anticoagulation in individuals with atrial fibrillation and cardiac amyloidosis, regardless of the CHADS-VASc score [88, 89].

An autopsy study done at the Mayo Clinic showed higher rates of intracardiac thrombi in patients with AL cardiac amyloidosis than with other types of cardiac amyloidosis [86]. Intracardiac thrombi were seen in all chambers of the heart. ATTR amyloidosis is also associated with a greater risk of intracardiac thrombus formation when compared to the general population [77, 90]. The incidence of intracardiac thrombi on transesophageal echocardiography (TEE) was also reported in individuals who had an onset of less than 48 hours of atrial fibrillation and individuals who were on systemic anticoagulation for 3 weeks prior to TEE, demonstrating the importance of performing a TEE prior to cardioversion in the cardiac amyloidosis population, regardless of prior anticoagulation [90].

There have been no prospective studies or randomized controlled trials comparing the efficacy and risk of the different anticoagulation agents such as direct oral anticoagulants (DOACs), vitamin K antagonists (VKAs) in atrial fibrillation in the cardiac amyloidosis population. A retrospective study by Habib *et al*. [91] utilizing an open access database in a population of 8214 individuals with ATTR cardiac amyloidosis demonstrated a lower incidence of ischemic stroke and a lower rate of intracranial hemorrhage with DOACs as compared to warfarin. No differences were detected in the two groups in terms of all-cause mortality, all-cause hospitalization, gastrointestinal hemorrhage, and hematuria. A smaller retrospective study of 273 patients which included individuals with different subtypes of cardiac amyloidosis showed a higher incidence of bleeding events in individuals treated with VKAs as compared to those treated with DOACs [92]. However, there was no difference in the incidence of ischemic stroke events, regardless of the amyloidosis subtype. The choice of anticoagulation agent remains an area of dispute and randomized controlled trials are needed before a robust recommendation is made.

Given the aforementioned challenges with pharmacologic therapy in this patient population, direct current cardioversion (DCCV) may be a desirable option, especially in symptomatic individuals and in those with hemodynamic instability. Reports of success and recurrence rates have been variable. El-Am *et al*. [90] found no difference in success and rates after DCCV between a cardiac amyloidosis population and a population without the diagnosis. Although the cardiac amyloidosis population experienced a higher rate of complications and DCCV was more likely to be cancelled, mainly due to the presence of an intracardiac thrombus on TEE. There was no difference in recurrence between the AL and ATTR amyloidosis subtypes. Sanchis *et al*. [62] reported a recurrence rate of 55% and 70% at 3 months, and 1 year, respectively [92]. Although recurrence at 1 year was not associated with a higher mortality risk. Regardless of anticoagulation status, DCCV should be preceded by a TEE in the individuals with cardiac amyloidosis due to the higher risk of intracardiac thrombi [90]. Data on the long-term recurrence remains lacking.

Data on catheter ablations for atrial arrhythmias in the cardiac amyloidosis population is scarce. A study reporting 18 catheter ablations in the cardiac amyloidosis population for atrial arrhythmias, showed a recurrence rate of 83% at 1 year while the recurrence rate in the control group was 14% [65]. Donnellan *et al*. [93] reported 24 catheter ablations for atrial fibrillation in patients with ATTR cardiac amyloidosis. They reported a recurrence rate of 58% with a mean follow up period of 39 months. Tan *et al*. [94] reported recurrence rates of 75% and 60% at 1 and 3-year intervals of atrial arrhythmias, respectively, in 26 patients with cardiac amyloidosis. Larger randomized controlled trials are needed to determine the efficacy of this rate control strategy.

### 5.2 Ventricular Arrhythmias 

The presence of premature ventricular complexes (PVCs), nonsustained ventricular tachycardia (NSVT), and sustained ventricular tachycardia (SVT) on cardiac monitoring is associated with sudden cardiac death [63, 95]. NSVT appears to be the most common ventricular arrhythmia with prevalence reported as 18%, 65%, and 74% in three different studies in patients with AL amyloidosis [63, 95, 96]. The presence of SVT and other forms of ventricular fibrillation has been noted to occur in 8%, and 19% in two different studies [63, 96]. Varr *et al*. [96] have considered the presence of NSVT an important factor to consider upon selecting patients for ICD devices in this patient population.

The cause of sudden cardiac death (SCD) in the cardiac amyloidosis patient population has been thought to be due to electromechanical dissociation leading to pulseless electrical activity (PEA) [97, 98]. Coupled with a higher defibrillation threshold thought to be refractory to ICD therapy [99], and poor prognosis, ICD implantation for primary and secondary prevention of SCD in this patient population has been an area of dispute in expert guidelines. The ESC cites insufficient data in their 2022 guidelines as the reason for the lack of recommendation of ICD as a means of primary prevention of SCD [69]. However, it provides a class IIa recommendation of ICD placement for secondary prevention of SCD in individuals with ventricular arrhythmias causing hemodynamic instability. An individualized approach for the placement of ICD for primary and secondary prevention of SCD in patients with cardiac amyloidosis is advised by the American Heart Association/American College of Cardiology/Heart Rhythm Society in their 2017 guidelines [68]. Both societies recommend against ICD placement for primary and secondary prevention of SCD in cardiac amyloidosis patients with a life expectancy of <1 year, in individuals who are not candidates for advanced therapies, and in patients with medication- refractory New York Heart Association class IV heart failure.

Despite similar rates of appropriate ICD therapies in the cardiac amyloidosis patient population to the rates reported in the DANISH and MADIT-II trials, no studies to date have shown a mortality benefit of ICD placement for primary or secondary prevention of SCD in patients with cardiac amyloidosis [74]. Lin *et al*. [100], found that 28% of patients with ICDs received appropriate shocks at 1 year in a population of 53 patients with cardiac amyloidosis. Most of the individuals who received appropriate shocks had ICDs placed for secondary prevention. There was no mortality benefit upon follow-up. Varr *et al*. [96] reported 26% of appropriate ICD shocks in 19 individuals with cardiac amyloidosis. None of the patients who received appropriate shocks had an ICD placed for primary prevention. A study by Hamon *et al*. [101] reported a 27% rate of appropriate shocks. Most ICDs (84%) placed were for primary prevention. Further data is required to demonstrate the benefit of ICD therapies in the cardiac amyloidosis population.

In conclusion, ventricular arrhythmias are frequent in cardiac amyloidosis and contribute significantly to the risk of sudden cardiac death, yet the benefit of ICD therapy in this population remains unclear. Current guidelines emphasize an individualized approach, generally reserving ICD placement for secondary prevention in patients with hemodynamic instability, while avoiding use in those with advanced heart failure or limited life expectancy. Although appropriate ICD shocks are commonly reported, no studies have demonstrated a definitive survival benefit to date.

### 5.3 Conduction Disease

Atrioventricular conduction delay is more prevalent than sinus node disease in the cardiac amyloidosis population [65, 66]. Reisinger *et al*. [64] demonstrated a prolonged infrahisian conduction time in 92% of patients with AL cardiac amyloidosis through electrophysiologic studies, whereas 88% had no sinus node disease. Although conduction disease is frequently seen in patients with this patient population, the mechanism behind it remains poorly understood [74]. In an autopsy study by Ridolfi *et al*. [102], 3 out of 23 patients had amyloid fibril deposition in conduction system tissue.

Permanent pacemakers (PPM) are frequently indicated in patients with cardiac amyloidosis and infrahisian conduction disease, especially in the ATTR subgroup with a study performed by Givens *et al*. [103] reported an incidence PPM implantation in 43% and 36% in patients with wtATTR and hATTR, respectively. There has been no evidence of the benefit of prophylactic pacing. As for cardiac resynchronization therapy, Donnellan *et al*. [104] showed that cardiac resynchronization therapy in patients with ATTR cardiac amyloidosis and an indication for a pacemaker is associated with improvements in mitral regurgitation, NYHA class, and left ventricular ejection fraction, when compared to right ventricular pacing.

## 6. Advanced Heart Failure Therapies in Patients With Cardiac Amyloidosis

Durable mechanical circulatory support (MCS) and left ventricular assist devices (LVAD) were developed as a bridge to heart transplant in patients with end stage heart disease, all while offering good quality of life with low rates of adverse events [105, 106, 107]. Their use in end stage cardiac amyloidosis patients has not been extensively explored. This is mainly due to biventricular involvement and the small left ventricular cavity size [108, 109, 110]. A study by Swiecicki *et al*. [109] identified 9 patients with end stage cardiac amyloidosis who received continuous LVAD over a 4-year period. The median survival for patients discharged from the hospital was 17.1 months [109]. Regardless of whether an LVAD or biventricular MCS device is used, the INTERMACS registry shows that the use of MCS in cardiac amyloidosis is associated with a higher rate of adverse events and lower rates of survival when compared to patients with dilated cardiomyopathies or nonamyloid restrictive cardiomyopathies [111, 112].

There is scarce yet encouraging data about heart transplant (HT) in patients with cardiac amyloidosis [113]. Current data shows that with appropriate selection [114], a median survival of 5 years in patients with AL cardiac amyloidosis treated with HT and ASCT; non inferior to other nonamyloid cardiomyopathies [115, 116, 117, 118, 119]. Recurrence can occur [120], and therefore, post-transplant surveillance with endomyocardial biopsies is essential to detect any recurrence of the disease in this population [121].

As for ATTR cardiac amyloidosis, HT remains the only sole treatment modality that offers a potential return to normal cardiac function [122]. A multicenter study based in the UK and Italy showed survival rates of 100%, 92%, and 90% at 1, 3, and 5 years, respectively, in patients with ATTR cardiac amyloidosis receiving cardiac transplant [122]. The rates reported are comparable to nonamyloid cardiomyopathies. The risk of recurrence in this patient population is low, owing to the slow amyloid fibril accumulation [111].

In conclusion, HT remains a robust therapeutic option for patients with end stage cardiac amyloidosis to restore normal heart function, improve survival, and restore quality of life.

## 7. The Management of Aortic Stenosis in Cardiac Amyloidosis

The coexistence of cardiac amyloidosis and aortic stenosis (AS) has recently been revealed itself to be more common than previously thought and carries a bad prognosis. Overall, ATTR is more commonly associated with cardiac amyloidosis, especially in older males (>70 years) with low flow AS [123, 124, 125, 126]. The data regarding the therapeutic management of aortic stenosis with coexisting cardiac amyloidosis is scarce. Most studies have shown a higher risk of mortality with aortic valve replacements in patients with cardiac amyloidosis [125, 127, 128, 129]. Furthermore, due to the fragile nature of amyloid infiltrated tissue, patients with cardiac amyloidosis are at higher risk of periprocedural and early postprocedural complications with transcatheter aortic valve replacement [130, 131]. Generally, a multidisciplinary team approach should be taken when discussing the treatment options in patients with coexisting AS and cardiac amyloidosis. Frailty, comorbidities, life expectancy, and functional status should be taken into account before proceeding with a certain treatment method. If aortic valve replacement is considered to be futile, medical therapy should be optimized. Otherwise, aortic valve replacement can be considered [132]. Factors such as decreased ejection fraction of less than 50%, severely reduced global strain of at least –10%, grade III diastolic dysfunction, a low flow state with a stroke volume index of <30 mL/m^2^, and low gradient AS are associated with poor prognosis and a futile attempt at aortic valve replacement, especially if the surgical option is considered [123, 129]. In case any of the factors are present on imaging, transcatheter aortic valve replacement (TAVR) is thought to be a more suitable option than surgical aortic valve replacement (SAVR) [132].

## 8. The Management of Orthostatic Hypotension in Cardiac Amyloidosis

Orthostatic hypotension (OH), which presents with symptoms of dizziness, fatigue, and syncope, is notorious for being difficult to treat and is common among patients with cardiac amyloidosis. It can occur due to amyloid cardiomyopathy, volume depletion, as a result medication adverse effects, or due to diarrhea. Reviewing medication lists is the first step in management. Alpha blockers, antidepressants with sympatholytic activity due to their alpha-2 activity, nitrates and diuretics are associated with worsening of orthostatic hypotension. The benefits and risks of these drugs need to be considered [133]. Caffeine and alcohol consumption should be reduced due to their diuretic effects. Cautiously increasing water and water intake in patients without volume overload can be considered. Eating small meals to avoid splanchnic dilation and physical exercise should be encouraged. Compression stockings can mobilize venous return to the heart and alleviate symptoms [133, 134].

Midodrine is considered the first line treatment for patients with cardiac amyloidosis who have orthostatic hypotension. It works as an alpha-1 receptor agonist and increases vascular tone. Droxidopa, a synthetic norepinephrine precursor, is another medication that can be used as an adjunct to midodrine. Fludrocortisone is another option that increases water retention by the kidneys but is associated with increased hospitalization when compared to midodrine [134, 135].

## 9. Conclusions

Cardiac amyloidosis has transitioned from a historically underdiagnosed and untreatable disease to a well-studied cardiomyopathy with an expanding spectrum of therapeutic options. Advances in noninvasive imaging and heightened clinical awareness have allowed earlier identification of both immunoglobulin AL and ATTR subtypes, enabling timely initiation of disease-modifying therapy and multidisciplinary management strategies.

For AL amyloidosis, the rapid suppression of light-chain production remains the cornerstone of treatment, with daratumumab-based regimens and ASCT markedly improving survival. For ATTR amyloidosis, transthyretin stabilizers (tafamidis, acoramidis), RNA-silencing therapies (patisiran, vutrisiran, eplontersen), and emerging CRISPR-based gene-editing strategies (NTLA-2001) are redefining long-term disease trajectories by addressing the root molecular mechanism of amyloidogenesis.

Arrhythmia management in cardiac amyloidosis remains particularly challenging due to restrictive physiology, electrical instability, and poor tolerance of conventional rate-control therapies. Beta blockers, non-dihydropyridine calcium channel blockers, and digoxin often exacerbate hypotension or conduction abnormalities. Consequently, rhythm-control strategies using amiodarone are favored, with maintenance of sinus rhythm shown to improve hemodynamics and functional status, especially in early-stage disease. Anticoagulation is mandatory in all amyloidosis patients with atrial fibrillation—regardless of CHA₂DS₂-VASc score—owing to the high risk of intracardiac thrombus formation even in sinus rhythm. Decisions regarding device implantation should be individualized with ICDs generally reserved for secondary prevention in patients with hemodynamically significant ventricular arrhythmias, while cardiac resynchronization therapy may provide symptomatic and functional improvement in selected patients requiring pacing.

Orthostatic hypotension, a common and debilitating manifestation, results from autonomic neuropathy. Its management relies on lifestyle interventions with pharmacologic therapy. Midodrine remains the first-line agent; droxidopa can be added in refractory cases, and fludrocortisone may be cautiously used to augment intravascular volume. AS frequently coexists with ATTR amyloidosis, particularly in older men with low-flow, low-gradient physiology. This overlap portends a worse prognosis and complicates treatment decisions. Recognition of dual pathology through careful imaging and multimodality assessment is crucial, as surgical risk is elevated and outcomes after valve intervention remain variable. When feasible, TAVR is often preferred over surgical replacement, especially in patients with preserved frailty and advanced amyloid infiltration. A multidisciplinary team approach is essential to individualize the decision-making process. Patients deemed unsuitable for intervention should receive optimized medical therapy and palliative support.

Ultimately, the management of cardiac amyloidosis requires a multidisciplinary approach encompassing disease-specific therapy, arrhythmia control, hemodynamic optimization, and supportive care. Continued research should focus on real-world outcomes, therapeutic accessibility, and the optimal sequencing or combination of emerging therapies. With the rapid evolution of targeted therapeutics, gene editing, and immunomodulation, cardiac amyloidosis is increasingly becoming a treatable cardiomyopathy.
